# Inducible Nitric Oxide Inhibitors Block NMDA Antagonist-Stimulated Motoric Behaviors and Medial Prefrontal Cortical Glutamate Efflux

**DOI:** 10.3389/fphar.2015.00292

**Published:** 2015-12-15

**Authors:** Hadley C. Bergstrom, Altaf S. Darvesh, S. P. Berger

**Affiliations:** ^1^Department of Psychology, Program in Neuroscience and Behavior, Vassar College, PoughkeepsieNY, USA; ^2^Department of Pharmaceutical Sciences, College of Pharmacy, Northeast Ohio Medical University, RootstownOH, USA; ^3^Department of Psychiatry, College of Medicine, Northeast Ohio Medical University, RootstownOH, USA; ^4^Department of Veterans Affairs Medical Center, PortlandOR, USA

**Keywords:** green tea, schizophrenia, phencyclidine, nNOS, iNOS, stereotypy, ataxia, microdialysis

## Abstract

Nitric oxide (NO) plays a critical role in the motoric and glutamate releasing action of *N*-methyl-D-aspartate (NMDA)-antagonist stimulants. Earlier studies utilized neuronal nitric oxide synthase inhibitors (nNOS) for studying the neurobehavioral effects of non-competitive NMDA-antagonist stimulants such as dizocilpine (MK-801) and phencyclidine (PCP). This study explores the role of the inducible nitric oxide synthase inhibitors (iNOS) aminoguanidine (AG) and (-)-epigallocatechin-3-gallate (EGCG) in NMDA-antagonist induced motoric behavior and prefrontal cortical glutamate eﬄux. Adult male rats were administered a dose range of AG, EGCG, or vehicle prior to receiving NMDA antagonists MK-801, PCP, or a conventional psychostimulant (cocaine) and tested for motoric behavior in an open arena. Glutamate in the medial prefrontal cortex (mPFC) was measured using *in vivo* microdialysis after a combination of AG or EGCG prior to MK-801. Acute administration of AG or EGCG dose-dependently attenuated the locomotor and ataxic properties of MK-801 and PCP. Both AG and EGCG were unable to block the motoric effects of cocaine, indicating the acute pharmacologic action of AG and EGCG is specific to NMDA antagonism and not generalizable to all stimulant class drugs. AG and EGCG normalized MK-801-stimulated mPFC glutamate eﬄux. These data demonstrate that AG and EGCG attenuates NMDA antagonist-stimulated motoric behavior and cortical glutamate eﬄux. Our results suggest that EGCG-like polyphenol nutraceuticals (contained in “green tea” and chocolate) may be clinically useful in protecting against the adverse behavioral dissociative and cortical glutamate stimulating effects of NMDA antagonists. Medications that interfere with NMDA antagonists such as MK-801 and PCP have been proposed as treatments for schizophrenia.

## Introduction

One important second messenger system involved in signal transduction for many neurotransmitters, including glutamate, is nitric oxide (NO; Garthwaite, 1991). Several groups have evaluated whether nitric oxide synthase (NOS) inhibition counteracts the behavioral effects of psychotomimetic *N*-methyl-D-aspartate (NMDA) subtype of glutamate receptor antagonists (Wiley, 1998; Bujas-Bobanovic et al., 2000). Studies using either non-specific or neuronal nitric oxide synthase (nNOS) inhibitors yielded conflicting results. Some studies showed that nNOS inhibitors, like glycine-type NMDA agonists, block the behavioral effects of phencyclidine (PCP)-like NMDA antagonists (Deutsch et al., 1996; Klamer et al., 2004). While others show enhanced responsiveness to PCP when pretreated with nNOS inhibitors (Bujas-Bobanovic et al., 2000). Subsequent studies utilizing nNOS gene deletion in knockout mice showed diminished responsiveness to PCP (Bird et al., 2001). Collectively, these studies established a causal role for nNOS in glutamate signaling. Relative to nNOS, few studies have examined a role for NO derived from the inducible isoform (iNOS) in glutamate signaling. This is presumably because iNOS is not enzymatically detectable in “naïve” animals and is mainly observed in the context of significant neuronal injury (Licinio et al., 1999; Cardenas et al., 2000; Amitai, 2010). Although confined to glial cells, iNOS, if activated by the neurotoxic action of NMDA receptor blockade would generate a readily diffusible gas capable of reaching behaviorally relevant neuronal sites (Olney et al., 1991).

Aminoguanidine (AG) has extensively been used for preferentially inhibiting iNOS expression in the brain (Iadecola et al., 1995; Corbett and McDaniel, 1996; Lecanu et al., 1998; Wada et al., 1998; Olivenza et al., 2000; Sugimoto and Iadecola, 2002; Harvey et al., 2004; Louin et al., 2006; Udayabanu et al., 2008). Another compound that inhibits iNOS expression is (-)-epigallocatechin-3-gallate (EGCG; Chan et al., 1997; Lin and Lin, 1997; Kim et al., 2010). EGCG is the major polyphenol in “green tea” and considered the central bioactive molecule responsible for the health benefits of green tea (Liao et al., 2001). Establishing a role for the iNOS inhibitors AG and EGCG in NMDA antagonist action is of significant clinical interest because NMDA medications have been proposed as treatments for schizophrenia (Neill et al., 2010).

In this series of experiments we evaluated the structurally unrelated iNOS inhibitors aminoguanidine and EGCG for their potential to block the potent motoric effects of the NMDA antagonist dizocilpine (MK-801) and PCP. Cocaine was included into the experimental design as a control for evaluating the generalizability of AG and EGCG on motoric behavior from all stimulants, regardless of mechanism. We hypothesized that iNOS inhibitors (AG and EGCG) attenuate the acute psychostimulant properties of the NMDA-antagonists MK-801 and PCP at doses that do not diminish cocaine-induced locomotion.

In addition to motoric behavior, NMDA receptor antagonists also mediate prefrontal glutamate release. Administration of MK-801 has been shown to elevate prefrontal glutamate (Moghaddam et al., 1997; Moghaddam and Adams, 1998; Lorrain et al., 2003) and PCP reduces prefrontal glutamate (Fattorini et al., 2008, 2009). No study to date has directly tested whether iNOS inhibition, as has earlier been reported for nNOS inhibition (Roenker et al., 2012), is sufficient to mediate MK-801-induced medial prefrontal cortical (mPFC) glutamate release. We hypothesized that AG and EGCG would inhibit MK-801-stimulated mPFC extracellular glutamate release as measured by *in vivo* microdialysis (Darvesh et al., 2011).

## Materials and Methods

### Animals

Experimentally naïve male Sprague–Dawley rats (Harlan; Indianapolis, IN, USA) were tested in all experiments. Rats averaged 70–100 days of age, weighed between 150–250 g at time of testing and were maintained on a 12:12 light:dark cycle (lights on at 0600) with an ambient temperature maintained at 21 ± 2°C. Food and water were available *ad libitum*. Animal husbandry was in accordance with the National Institutes of Health Guidelines for the Care and Use of Laboratory Animals (2011) and approved by the Portland Veterans Affairs Institutional Animal Care and Use Committee (IACUC). Disclosure of housing and husbandry procedures was in accordance with recommendations for standard experimental reporting in behavioral neuroscience research (Prager et al., 2011).

### Drugs

Dizocilpine maleate ((+)-MK-801), phencyclidine hydrochloride (PCP), and cocaine hydrochloride (COC) were obtained from Research Biochemicals International (RBI; Natick, MA, USA). (-)-Epigallocatechin gallate (EGCG) and aminoguanidine hydrochloride (AG) were obtained from Sigma (St Louis, MO, USA). All drugs were diluted to appropriate concentrations in physiological saline (Abbott laboratories; North Chicago, IL, USA). Injection volume was 0.1 ml 100g^-1^ body weight for all drug-treated rats, and for vehicle.

### Apparatus

Rats were tested in automated locomotor activity monitors (Accuscan Instruments Inc. Columbus, OH, USA). Each test chamber comprised a clear acrylic plastic box (43 cm wide x 43 cm long x 20 cm tall; 17 x 17 x 8) with a clear plastic lid (47 cm x 47 cm) perforated for ventilation. Each box was placed inside a 43 cm x 43 cm square monitor, with 15 photocell beams equally spaced (1″ on center) on each axis at right angles to one another, 2 cm above the floor. Each monitor and box sat inside a sound-dampening, light-proof cabinet. The inside was illuminated by a 25-watt halogen light during testing. Internal fans provided ventilation and a fixed level of background noise.

### Schedule and Procedure

#### Experiment 1: AG and NMDA-antagonist-induced motoric Behavior

On test day, rats were transferred in the home cage to the testing room and allowed to habituate for 45–60 min Rats received intraperitoneal (i.p.) injections of AG (40, 100, 400 mg/kg) or saline (vehicle) and placed into the activity chambers for 30 min. The dose range for i.p. administration of AG was based on several previous reports (Olivenza et al., 2000; Louin et al., 2006). The initial 30 min period allowed for habituation to the novel environment. Upon completion of the 30 min interval, the animals were removed from the chambers, placed in their home cages and, depending on group assignment, injected with MK-801 (MK; 0.25 mg/kg), PCP (20 mg/kg), cocaine (COC, 30 mg/kg), or an equal volume of saline vehicle (SAL). Immediately following injection, rats were transferred back into their original testing chambers for a 60 min test session. Previous studies suggest a 60 min window provides ample time for the full expression of the locomotor stimulant effects of MK-801, PCP, and COC (Diana and Sagratella, 1994; Hargreaves and Cain, 1995). Data were collected in 1-min time periods and quantified in 5-min bins. DRUG 1 (iNOS inhibitor or vehicle) was followed 30 min later by DRUG 2 (NMDA antagonist, cocaine, or vehicle), resulting in the following groups: iNOS inhibitor/NMDA antagonist, vehicle/NMDA antagonist, iNOS inhibitor/vehicle, vehicle/vehicle.

#### Experiment 2: AG and NMDA Antagonist-induced Ataxia and Stereotypy

A possible explanation for any effects of AG on NMDA-antagonist-induced locomotor behavior is that iNOS inhibitors shift the behavioral dose response curve leftward, increasing overall responsiveness. As the dose of the stimulant increases, motoric behaviors may decrease due to an increase in competing stereotypic or ataxic behaviors. Therefore, a reduction in locomotor activity due to iNOS inhibition could have been due to an increase in stereotypic and/or ataxic behavior. To test this possibility, ataxic and stereotypic behaviors in a separate group of rats was measured in the novel open field (same apparatus as above experiments) using a dosing schedule identical to that used for the locomotor experiments. Rats were administered SAL or AG (100 mg/kg) and placed into the activity chamber for 30 min. Then, rats were administered either SAL or MK, and placed into the chambers for 60 min. Rats were rated for ataxic and stereotypic behaviors using a well-validated rating scale adapted from (Sturgeon et al., 1979) (see Supplemental Material). This rating scale has been used for quantifying PCP-induced stereotypy and ataxic behavior in rats. All ratings were conducted by trained experimenters who scored the behavior of each rat for each of the two scales for 15–20 s at 10 min intervals over 60 min. Locomotor data was simultaneously collected. PCP was not evaluated for ataxic and stereotypic behaviors because PCP and MK-801 are both classified as non-competitive NMDA antagonists and produce relatively similar effects on motoric, stereotypic, and ataxic behaviors (Koek et al., 1988; Hiramatsu et al., 1989). We did not evaluate cocaine because we did not find effects of AG on cocaine-induced locomotor activity.

#### Experiment 3: EGCG and NMDA Antagonist-induced Motoric Behavior

Since iNOS is not constitutively present in the brain this raises the question of why an iNOS inhibitor would be behaviorally active. One possible explanation would be that AG has behavioral effects by an iNOS independent mechanism. We therefore examined whether EGCG (10, 30, 100 mg/kg), a structurally unrelated iNOS inhibitor, had comparable behavioral effects using a dosing and behavioral protocol identical to that described above for AG. The dose range for i.p. administration of EGCG was based on several previous reports (Vignes et al., 2006; Ge et al., 2013). Because AG was found to diminish stereotypic and ataxic behavior after MK-801, stereotypy and ataxia was not characterized following EGCG/MK-801.

#### Experiment 4: *In Vivo* Microdialysis of Cortical Glutamate

To our knowledge, no study to date has measured MK-801-induced mPFC extracellular glutamate release in the presence of an iNOS inhibitor. Rats were implanted with a stainless steel guide cannula under isoflurane anesthesia three days prior to insertion of the microdialysis probe. On the day prior to the experiment, a concentric style dialysis probe was positioned in the mPFC. The coordinates for the probe tip were AP: +3.2 mm, ML: +0.5 mm, DV: –5.0 mm from the bregma (Paxinos and Watson, 1986). The length of the probe membrane was 3.0 mm. The probe was connected to an infusion pump set to deliver aCSF (in mM: 140 NaCl, 3.4 KCl, 1.5 CaCl_2_, 1.0 MgCl_2_, 1.4 NaH_2_PO_4_, 4.85 NaHPO_4_, pH 7.4). The aCSF was allowed to flow through the probe overnight at a flow rate of 0.2 μl/min. On the morning of the experiment, the flow rate was increased to 2.0 μl/min and after 1 h equilibration period, dialysis samples were collected every 15 min for 4 h. Glutamate levels were measured by HPLC analysis (Donzanti and Yamamoto, 1988). Based on the experiments described above showing the lowest effective dose ranges for blocking MK-801-induced locomotor behavior, rats were injected (i.p.) with AG (100 mg/kg), EGCG (100 mg/kg), or vehicle (saline) 30 min prior to an injection of MK-801 (0.30 mg/kg) or vehicle. The slightly higher dose of MK-801 used in the microdialysis experiment (0.30 mg/kg) versus locomotor activity experiments (0.25 mg/kg) was based on a similar previous *in vivo* microdialysis study of MK-801-induced glutamate release and NO inhibition in the rat prefrontal cortex (Roenker et al., 2012). PCP and cocaine were not included into the experimental design as the effects of iNOS inhibition on PCP were comparable with MK-801 and iNOS was ineffective in blocking cocaine-induced locomotion.

### Data Analyses

For locomotor activity, the dependent variable was mean beam breaks in the open field. For ataxic and stereotopic behavior, the dependent variable was the mean ataxia or stereotypy rating on a 0–5 scale (Sturgeon et al., 1979) (see Supplemental Materials). For the microdialysis experiments, glutamate measures were transformed to a percent of mean baseline value (% baseline). All group comparisons of behavior were conducted using one-way analysis of variance (ANOVA) with DRUG (iNOS inhibitor, stimulant, iNOS inhibitor/stimulant, saline) defined as the between-groups factor. Homogeneity of variance was checked using Levene’s test and corrected if warranted using the Brown–Forsythe *F*. Follow-up *post hoc* comparisons (Fisher’s LSD *post hoc*) were only performed subsequent to a statistically significant ANOVA test. Time course locomotor and microdialysis glutamate measures across the testing sessions was conducted using ANOVA with repeated measures on the variable TIME. Significant interactions were followed up with post hoc comparisons (Fisher’s LSD *post hoc*). Because the saline/saline (saline) and saline/MK-801 (MK-801) groups were the only groups common across all experiments, a smaller number of subjects were run in each experiment (*n* = 3–4) and then collapsed across experiments to reduce the overall number of animals needed for experimentation. A *p*-value of at least 0.05 was considered significance for all statistical tests. All data are presented as mean ± SEM and sample size for each experiment is indicated in the figure legends.

## Results

### Aminoguanidine Blocks MK-801-induced Locomotor Activity

**FIGURE 1:**
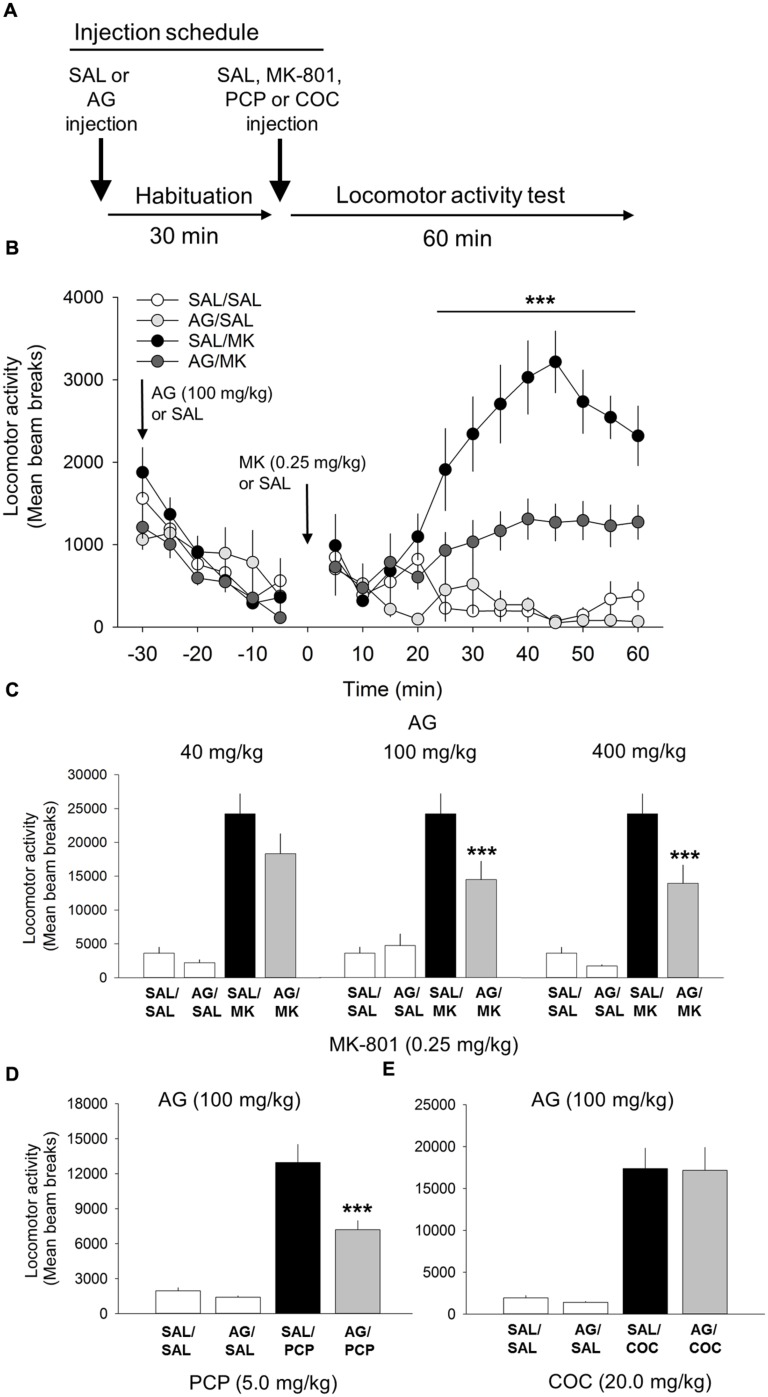
**Aminoguandine (AG) attenuates the locomotor stimulant properties of MK-801 and phencyclidine (PCP).** **(A)** Schematic depicting the schedule of injections and locomotor testing. Rats were injected (i.p.) with either saline vehicle (SAL) or AG (40, 100, or 400 mg/kg) 30 min prior to SAL or MK-801 (MK; 0.25 mg/kg), PCP (5.0 mg/kg) or cocaine (COC; 20.0 mg/kg). **(B)** Time-course analysis of locomotor activity following injections of AG (100 mg/kg), MK-801 or a combination of AG and MK-801. Attenuation of MK-801-induced locomotion by AG began rapidly, 15 min post injection, and persisted throughout the 60 min time window. **(C)** The attenuating effects of AG on MK-801 induced locomotor behavior in the open field were dose-dependent with 40 mg/kg having no effect and 100 and 400 mg/kg potently attenuating MK-801 locomotion. **(D)** AG attenuated PCP-induced locomotion. **(E)** AG did not attenuate the psychomotor stimulant action of cocaine. *n* = 9–20/group. ^∗∗∗^indicates *p* < 0.001 MK/AG compared to MK group. Data points represent mean ± SEM.

One-way ANOVA revealed a significant interaction of the iNOS inhibitor AG and MK-801 on motoric behavior [*F*(5,55) = 17.3, *p* < 0.001; **Figure 1**]. A *post hoc* test showed that MK-801 (0.25 mg/kg) potently facilitated locomotor activity compared to the vehicle control (*p* < 0.001) and AG by itself (*p* < 0.001). AG significantly blocked the locomotor effects of MK-801 at the 100 mg/kg (*p* < 0.001) and 400 mg/kg (*p* < 0.001) dose, but not 40 mg/kg dose (**Figure 1**). The attenuating effects of AG on MK-801-induced locomotor activity at the 100 and 400 mg/kg dose were statistically indistinguishable from each other (*p* = 0.15). A time course analysis of AG at the 100 mg/kg dose with DRUG as the between-subjects factor and TIME the within-subject factor revealed a significant interaction of DRUG x TIME [*F*(33,407) = 10.3; *p* < 0.001; **Figure 1**]. Attenuation of MK-801-induced locomotion by AG began 15 min post injection (*p* < 0.05) and persisted throughout the 60 min time window (*p* < 0.001; **Figure 1**). There were no significant differences detected between-groups prior to injection of MK-801. We also found no significant effects of various concentrations of AG on their own after vehicle injection (**Figure 1**). Time course analysis of AG/MK-801 at the 40 and 400 mg/kg dose not shown. Overall, these results suggest that the iNOS inhibitor AG is capable of significantly reducing NMDA-antagonist mediated motoric behavior. These data also suggest that AG may have an effect on the enzymatic function of iNOS.

### Aminoguanidine Attenuates PCP-induced Locomotor Activity

One-way ANOVA revealed a significant interaction of AG and PCP on motoric behavior [*F*(3,38) = 51.2, *p* < 0.001; **Figure 1**]. PCP treatment significantly increased locomotor activity compared to vehicle and AG alone at the 20 mg/kg dose (*p* = 0.001). AG (100 mg/kg) significantly reduced the locomotor effects of PCP (*p* < 0.001). This result further supports the conclusion that iNOS inhibition via AG attenuates NMDA-antagonist mediated motoric behavior.

### Aminoguanidine does not Alter Cocaine-induced Locomotor Activity

Cocaine was included into the experiment to assess the specificity of AG on the locomotor stimulant properties of NMDA receptor blockade. Results from this experiment showed that AG was unable to block the motoric effects of cocaine (20 mg/kg; **Figure 1**), indicating that iNOS inhibition is specific to NMDA-mediated locomotor activity and not general to all stimulant classes of drugs.

### Aminoguanidine Attenuates MK-801-induced Ataxia/Stereotypy

**FIGURE 2:**
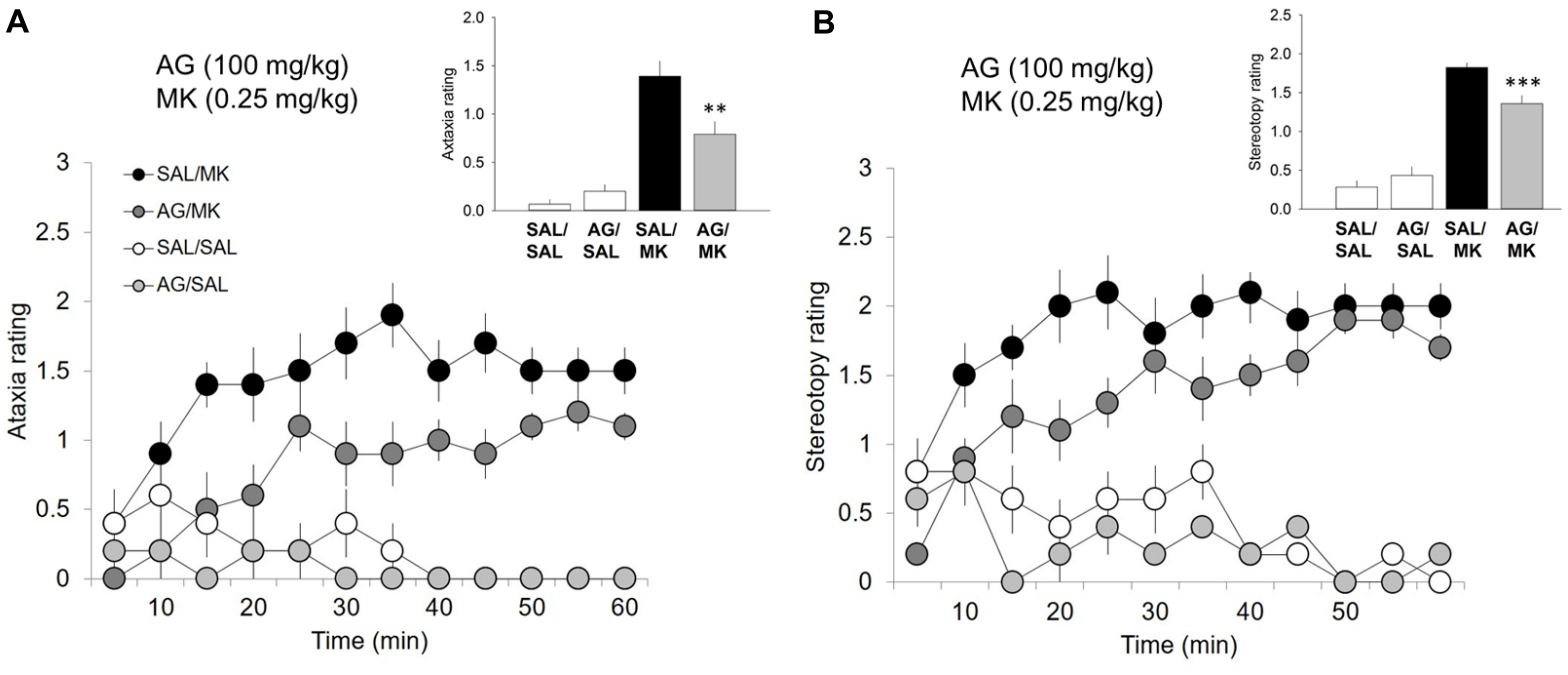
**Aminoguanidine attenuates MK-801-induced ataxic and stereotypic behavior.** Time-course ataxia and stereotypy measures in the open field following injections of AG (100 mg/kg), MK-801 (MK; 0.25 mg/kg), saline (SAL), or a combination of AG/MK-801. Rats were injected (i.p.) with either SAL or AG (100 mg/kg) 30 min prior to SAL or MK. **(A)** AG in the presence of MK-801 attenuated ataxic behavior. **(B)** AG in the presence of MK-801 attenuated ataxic behavior. *n* = 5–10/group. ^∗∗∗^*p* < 0.001 and ^∗∗^*p* < 0.01 AG/MK compared to MK group. Data points represent mean ± SEM. Ataxia and stereotypy rating scale adapted from (Sturgeon et al., 1979; see Supplemental Material).

One-way ANOVA revealed a significant interaction of the iNOS inhibitor AG and MK-801 on ataxic [*F*(3,26) = 18.1; *p* = 0.001; **Figure 2**] and stereotypic [*F*(3,26) = 62.0, *p* < 0.001; **Figure 2**] behavior. MK-801 significantly increased both ataxic and stereotypic behavior compared to AG (*p* < 0.001) and vehicle (*p* < 0.001). AG significantly reduced the ataxic (*p* < 0.01) and stereotypic (*p* < 0.001) inducing effects of MK-801. This result indicates that the ability of AG to attenuate the stimulant effects of MK-801 is not a result of increases in competing (ataxic or stereotypic) behaviors. MK-801 stimulated locomotor, ataxic and stereotypic behavior is consistent with previous reports.

### EGCG Blocks MK-801-induced Locomotor Activity

**FIGURE 3:**
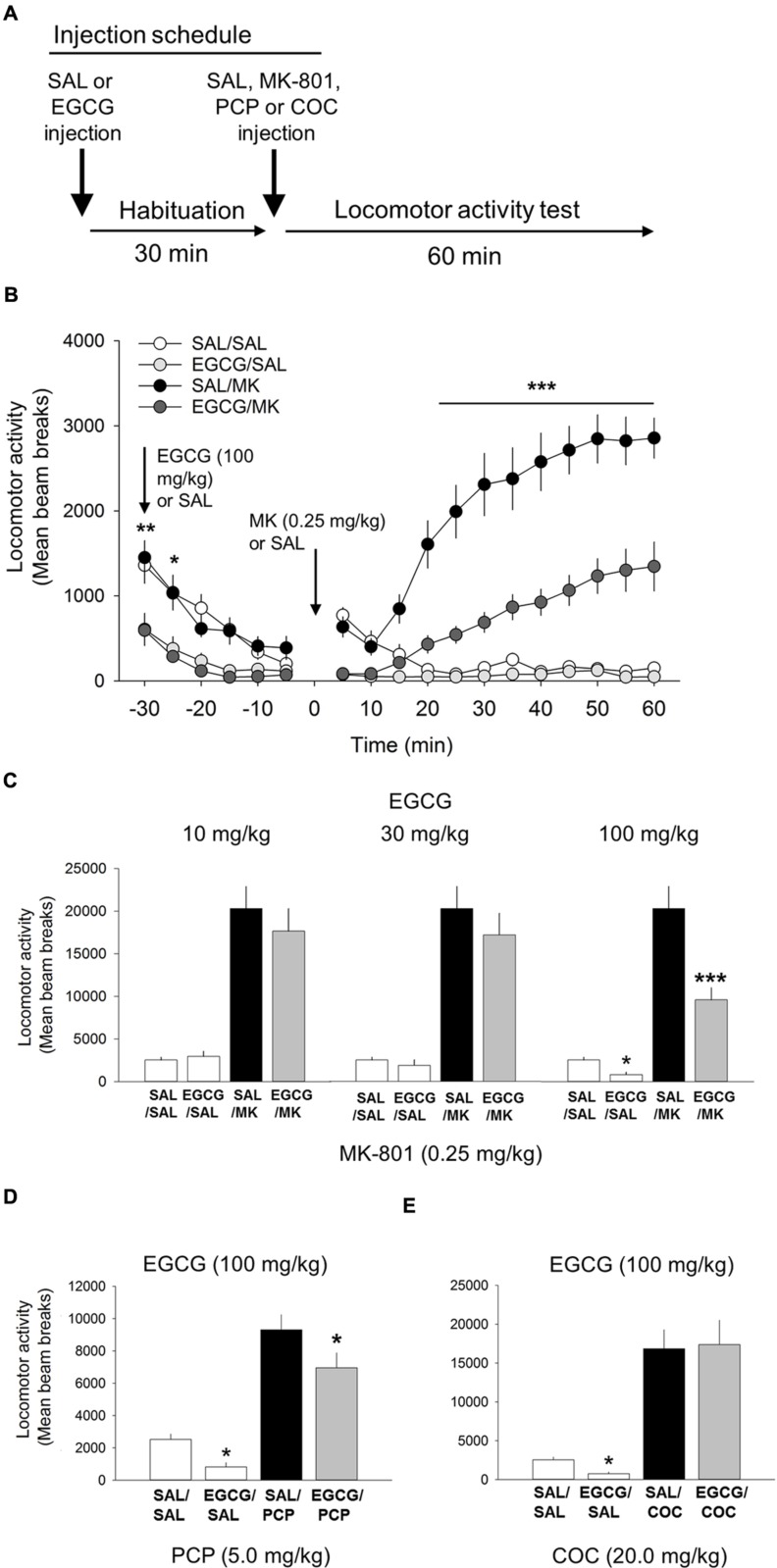
**(-)-Epigallocatechin-3-gallate (EGCG) attenuates the psychomotor stimulant action of MK-801 and PCP.** **(A)** Schematic depicting the schedule of injections and locomotor testing. Rats were injected (i.p.) with either vehicle (saline) or EGCG (10, 30, or 100 mg/kg) 30 min prior to vehicle saline (SAL) or MK-801 (MK; 0.25 mg/kg). **(B)** Time-course locomotor activity following injections of EGCG (100 mg/kg), MK-801 or a combination of EGCG and MK-801. Beam breaks due to locomotor activity are shown in 5-min epochs. Attenuation of MK-801-induced locomotion by EGCG (100 mg/kg) began rapidly, approximately 25 min post injection, and persisted throughout the 60 min time window. There was also a small, but significant decrease in locomotor activity following EGCG alone compared with saline control. **(C)** The attenuating effects of EGCG on MK-801 induced locomotor behavior were dose-dependent with the 10 and 30 mg/kg doses having no effect but 100 mg/kg attenuating MK-801-induced locomotion. EGCG by itself was also found to significantly reduce locomotor behavior relative to vehicle control. **(D)** EGCG was also able to attenuate PCP-induced locomotion. **(E)** EGCG was unable to attenuate the potent locomotor effect of cocaine (30 mg/kg). *n* = 9–21/group. ^∗∗∗^*p* < 0.001 EGCG/MK compared to MK group and ^∗^*p* < 0.05 and ^∗∗^*p* < 0.01 EGCG compared to SAL. Data points represent mean ± SEM.

We evaluated whether EGCG, another iNOS inhibitor structurally unrelated to AG, possessed comparable behavioral effects on NMDA-mediated locomotor behavior (**Figure 3**). Consistent with the AG studies, one-way ANOVA revealed a significant interaction of EGCG and MK-801 on motoric behavior [*F*(5,81) = 22.2; *p* < 0.001; **Figure 3**]. EGCG treatment prior to 0.25 mg/kg MK-801 reduced locomotor behavior at the 100 mg/kg (*p* < 0.001) but not 30 (*p* = 0.08) or 10 mg/kg dose (**Figure 3**). A time course analysis with DRUG as the between-subjects factor and TIME the within-subject factor revealed a significant interaction of DRUG x TIME [*F*(33,649) = 18.8; *p* < 0.001; **Figure 3**]. Attenuation of MK-801-induced locomotion by EGCG (100 mg/kg) began approximately 25 min post injection (*p* < 0.001) and persisted throughout the 60 min time window (**Figure 3**). Interestingly, EGCG by itself significantly reduced locomotor behavior relative to the saline control at 5 min (*p* < 0.001) and 10 min (*p* < 0.05) post injection (**Figure 3**). These data suggest that EGCG possesses comparable inhibitory effects on MK-801-induced locomotor behavior as the structural unrelated iNOS inhibitor AG. Overall, these results suggests that EGCG is capable of significantly reducing NMDA antagonist-mediated locomotor behavior.

### EGCG Attenuates PCP-induced Locomotor Activity

One-way ANOVA revealed a significant interaction of EGCG and PCP on motoric behavior [*F*(3,38) = 32.7, *p* < 0.001; **Figure 3**]. 100 mg/kg EGCG significantly decreased locomotor activity induced by 20 mg/kg PCP (*p* < 0.05). These data strengthen the assertion that EGCG lowers NMDA antagonist-mediated locomotor activity.

### EGCG does not Alter Cocaine-induced Locomotor Activity

Like the AG experiment described above, cocaine was included in the experiment to assess the specificity of EGCG on the locomotor stimulant properties of NMDA-anatagonists. Results from this experiment showed that EGCG was unable to block the motoric effects of cocaine (20 mg/kg; **Figure 3**). Overall, these data show that although EGCG effectively inhibited both psychotomimetics, MK-801, and PCP, it did not inhibit the non-NMDA psychomotor stimulant cocaine.

### MK-801 Induces mPFC Glutamate Eﬄux

**FIGURE 4:**
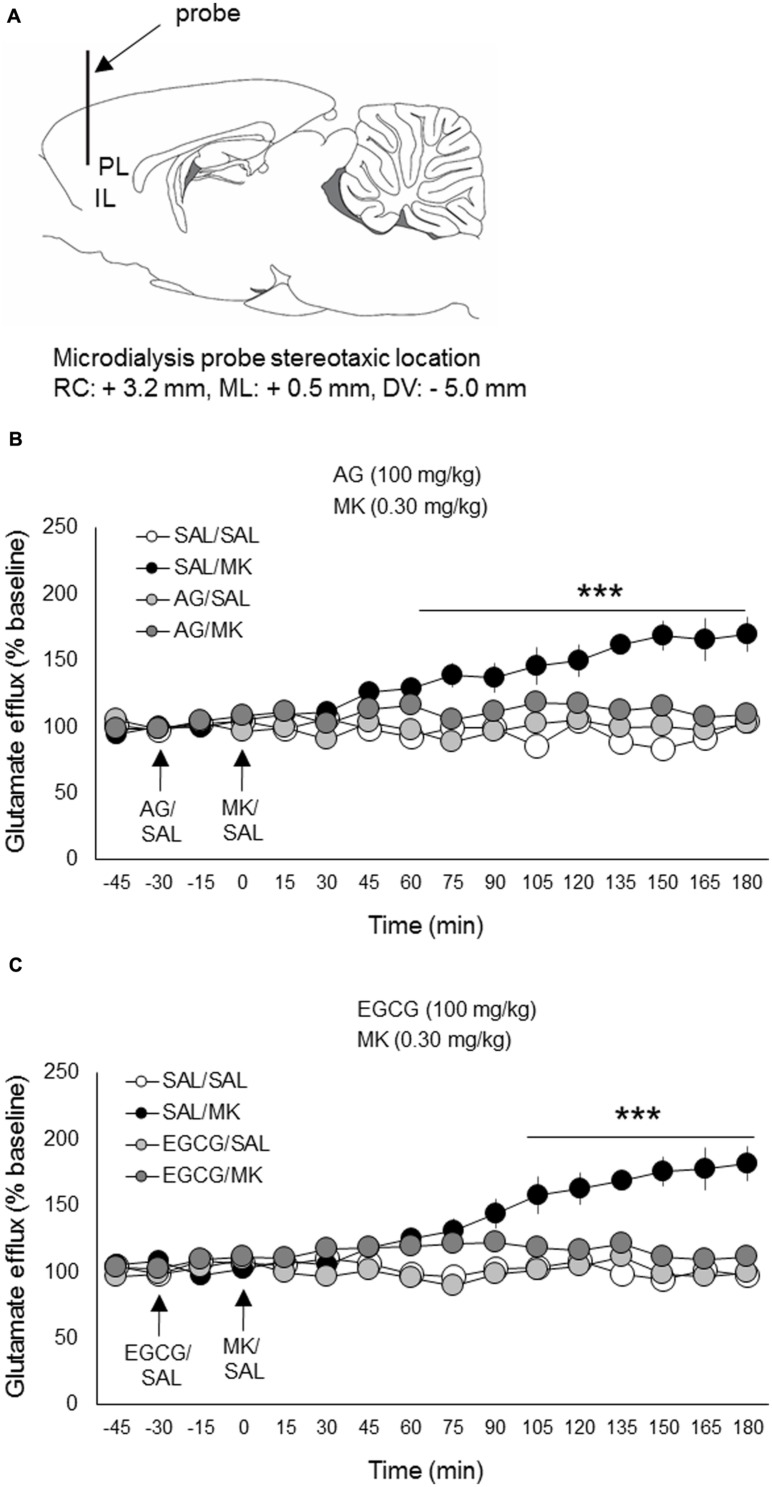
**Aminoguanidine and EGCG normalize MK-801 stimulated glutamate eﬄux in the prefrontal cortex.** Rats were injected (i.p.) with either AG (100 mg/kg, i.p.), EGCG (100 mg/kg), or vehicle (saline) 30 min prior to MK-801 (0.30 mg/kg, i.p.) or vehicle. **(A)** Schematic depicting the stereotaxic location of the microdialysis probe in the mPFC (PL/IL boundary). **(B)** Pretreatment with AG reduced MK-801 stimulated mPFC glutamate release beginning 75 min post-injection. **(C)** Pretreatment with EGCG reduced MK-801 stimulated mPFC glutamate release beginning approximately 90 min post-injection. *n* = 6–8/group. PL, prelimbic cortex; IL, infralimbic cortex. ^∗∗∗^*p* < 0.001 EGCG/MK or AG/MK compared to MK group. Data points represent mean ± SEM.

The NMDA receptor antagonist MK-801 significantly elevated extracellular glutamate levels in the mPFC relative to vehicle, a finding consistent with previous work (Pietraszek et al., 2009; Roenker et al., 2011, 2012). MK-801 increased glutamate levels approximately 180% over baseline measures (**Figure 4**). This increase is also consistent with previous work using the same MK-801 dose (0.30 mg/kg i.p.; Roenker et al., 2012).

### Aminoguanidine Normalizes MK-801-induced mPFC Glutamate Eﬄux

The iNOS inhibitor AG (100 mg/kg) was injected (i.p.) 30 min prior to MK-801 (0.30 mg/kg). A time course analysis of extracellular glutamate levels with DRUG as the between-subjects factor and TIME the within-subject factor revealed a significant interaction of DRUG x TIME [*F*(33,308) = 12.8; *p* < 0.001]. Subsequent *post hoc* testing revealed that AG significantly lowered MK-801-induced glutamate levels 75 min following MK-801 injection and this reduction persisted throughout the testing session (180 min total; **Figure 4**). AG injections alone had no significant effect on extracellular glutamate levels relative to the vehicle condition. There were no pre-treatment effects detected.

### EGCG Blocks MK-801-stimulated Glutamate Release

The iNOS inhibitor EGCG (100 mg/kg) was injected (i.p.) 30 min prior to MK-801 (0.30 mg/kg). A time course analysis of extracellular glutamate levels with DRUG as the between-subjects factor and TIME the within-subject factor revealed a significant interaction of DRUG x TIME [*F*(33,312) = 14.2; *p* < 0.05]. Subsequent *post hoc* testing revealed that EGCG significantly attenuated MK-801-induced glutamate levels 90 min following MK-801 injection and this reduction persisted throughout the testing session (180 min total; **Figure 4**). EGCG injections alone had no significant effects on extracellular glutamate levels relative to vehicle control. There were no pre-treatment effects detected.

## Discussion

Consistent with our initial hypothesis, the iNOS inhibitors AG and EGCG dose-dependently attenuated the potent acute motor stimulatory effects of the non-competitive NMDA antagonists MK-801 and PCP at doses that did not diminish cocaine-induced locomotion. MK-801 and PCP-induced ataxia and stereotypy were also reduced by AG and EGCG, suggesting the psychomotor attenuating effects of AG and EGCG could not be attributed to an increase in competing (ataxic or stereotypic) behaviors. Also consistent with our initial hypothesis, both AG and EGCG normalized MK-801-stimulated extracellular glutamate levels in the mPFC. These are the first reported preclinical findings implicating AG and EGCG in both the motoric and glutamate releasing action of NMDA antagonist stimulants.

Results from these experiments are comparable to the results from studies assessing the efficacy of known antipsychotics on NMDA-antagonist-induced locomotor activity and cortical glutamate eﬄux. Clozapine and haloperidol were both found to attenuate MK-801-induced glutamate release in the mPFC (Lopez-Gil et al., 2007). Typical and atypical antipsychotics have also been demonstrated to attenuate the hyperlocomotor action of MK-801. Prazosin (Mathe et al., 1996), risperidone (Su et al., 2007), zotepine (Gattaz et al., 1994), haloperidol (Hoffman, 1992), and clozapine (Hoffman, 1992) all have been shown to attenuate the locomotor stimulant action of MK-801. The present findings may have clinical implications because the neurobehavioral action of NMDA-antagonists models several dimensions of schizophrenia pathology and symptomology (Javitt and Zukin, 1991; Neill et al., 2010).

Interestingly, EGCG at the 100 mg/kg (but not 10 and 30 mg/kg) dose produced a small but significant reduction in general locomotor activity compared with vehicle control, suggesting EGCG by itself may reduce spontaneous motoric behavior. To our knowledge, an interaction of acute EGCG and spontaneous locomotor activity in rats has not been previously shown. It seems unlikely that the attenuating effects of EGCG on NMDA-antagonist-induced locomotor stimulation can solely be accounting for by non-specific motor effects since EGCG produced no statistically significant effects on cocaine-induced locomotor activity. Another interesting interpretation for locomotor attenuation following EGCG is the possibility it reflects an acute anxiogenic property. This interpretation, however, is inconsistent with other data demonstrating EGCG induces anxiolytic responsivity on the elevated plus maze (Vignes et al., 2006), reverses the anxiogenic action of caffeine (Park et al., 2010) and reverses stress-induced decreases in locomotor activity (Soung et al., 2015). AG has also been shown to reverse anxiogenic and depressive behaviors following social isolation stress (Amiri et al., 2015). Finally, local injection of the more selective iNOS inhibitor 1400 W in the dorsolateral periaqueductal gray attenuated the anxiogenic action of ethanol withdrawal (Bonassoli et al., 2013). However, these pharmacological findings are in direct conflict with data generated using molecular genetic tools. INOS knock out mice were found to exhibit anxiogenic-like behavioral responsivity (Buskila et al., 2007), elevated stress reactivity (Abu-Ghanem et al., 2008) and greater contextual fear memory (Lisboa et al., 2015). Taken together, results from iNOS deletion mice suggest any putative anxiolytic action of EGCG may be iNOS-independent. This interpretation is supported by the present data showing the iNOS inhibitor AG by itself produced no effects on motoric behavior.

There is also some evidence that iNOS inhibition impacts cognition. EGCG has been shown to rescue cognitive deficits in a Down’s syndrome mouse model (De la Torre et al., 2014), although see (Stringer et al., 2015). INOS inhibition has been found to decrease learning and memory impairments in spontaneous hypertension rats (Wang et al., 2014). EGCG was also able to slow cognitive degeneration in mouse models of Alzheimer’s disease (Rezai-Zadeh et al., 2008; Chang et al., 2015). Finally, EGCG has been shown to improve spatial memory (Haque et al., 2006). Together, these studies indicate some cognitive improvement after iNOS inhibition via EGCG.

Our findings should be interpreted with caution. Many of the agents which interact with iNOS also modulate other inflammatory and non-inflammatory mechanisms such as the enzyme semicarbazide-sensitive amine oxidase and hormones such as testosterone and insulin (Kao et al., 2000). It is also possible that the pharmacologic action of AG and EGCG on NMDA-mediated motoric behavior and cortical glutamate eﬄux was not specific to iNOS. EGCG has been shown to suppress nNOS expression (Wei et al., 2011), COX-2 activity (Surh et al., 2001; Ahmed et al., 2002), JAK/STAT1 signaling (Giunta et al., 2006), DYRK1A expression (De la Torre et al., 2014), spontaneous excitatory synaptic (Vignes et al., 2006) and NF-κB activation (Surh et al., 2001). Future studies using iNOS gene knock-out mice, more specific iNOS inhibitors such as 1400 W, or measurements of iNOS expression following EGCG and AG administration are warranted to definitively conclude the relative contribution of iNOS to NMDA-mediated motoric behavior and cortical glutamate eﬄux. Nevertheless, the present series of experiments establish that AG and EGCG, two structurally unrelated iNOS inhibitors, robustly reduce NMDA-antagonist induced motoric behavior and cortical glutamate eﬄux.

### iNOS Induction and Expression

In the adult brain, excess NO has been classically associated with inflammation, injury or trauma. The inducible isoform of NO synthase may play a role in the persistence or even elevation of NO levels (Licinio et al., 1999; Heneka and Feinstein, 2001). INOS has been shown to be expressed in various brain regions following injury including the cortex (Olivenza et al., 2000; Madrigal et al., 2001; Buskila and Amitai, 2010), the hippocampus (Petrov et al., 2000), and striatum (Lecanu et al., 1998; Weldon et al., 1998). To our knowledge, no studies have examined the pattern of iNOS expression in the brain following acute or chronic NMDA antagonism. Our data indirectly suggest MK-801 and PCP induces iNOS. However, as discussed above, AG and EGCG exert multiple iNOS-independent effects.

### Schizophrenia and iNOS Inhibition

It has widely been suggested that the behavioral sequela associated with NMDA-antagonists may model schizophrenia more closely than amphetamine-like stimulants. These findings are similar to earlier studies of glycine-type NMDA agonists that also significantly reversed motoric, stereotypic, and ataxic effects of NMDA antagonists (Contreras, 1990). Of interest, these studies were the basis of successful double-blind studies of glycine-type NMDA agonists in schizophrenia. iNOS inhibitors may likewise merit study. Indeed, minocycline is one medication known to inhibit iNOS and has already been reported to be beneficial (mainly on negative symptomatology) in many studies on schizophrenia (Zhang et al., 2007). If minocycline exerts its effects by an iNOS mechanism, more specific iNOS inhibitors, such as AG or 1400W, may be even more therapeutically efficacious.

Minocycline was studied in schizophrenia based on preclinical studies similar to our own. For example, one study found minocycline pretreatment blocked MK-801 induced hyperlocomotion, prepulse inhibition of startle (PPI) and striatal/cortical elevations of dopamine as detected by microdialysis (Zhang et al., 2007). The authors attribute these findings to minocycline’s ability to block microglial activation without specifically discussing the resulting inhibition of iNOS.

### iNOS Inhibitors in the Emergency Room, Hospital Ward, and in Outpatients

These preliminary results may have clinical relevance for Emergency room medical staff management of PCP-intoxicated patients. These patients are among the most dangerous in psychiatry, because their agitation often culminates in delirium. Sadly, the response to antipsychotic medications is often inadequate. iNOS inhibitors merit study in the many dangerous PCP-intoxicated delirious patients refractory to conventional antipsychotics.

Phencyclidine-like delirium is often observed in patients hospitalized for a variety of medical conditions. Most of these conditions likely elicit sufficient inflammation to induce iNOS in the CNS or periphery. Like PCP-intoxicated patients, such patients are often refractory to high doses of dopamine antagonists. Future study is warranted to determine whether PCP-intoxicated patients and patients with PCP-like delirium benefit from iNOS inhibitors.

Finally, the dissociative medication ketamine is often used as an anesthetic in an emergency room setting and as a rapidly acting antidepressant (Abdallah et al., 2015) in depressed outpatients participating in research studies. Our preclinical study raises the possibility of iNOS inhibitors preventing aversive side effects of ketamine-like medications. However, there is an important caveat. Little is known about how iNOS inhibitors would interact with the therapeutic effects of ketamine-like anesthetics and antidepressants.

## Conclusion

*N*-methyl-D-aspartate antagonist compounds, despite significant adverse dissociative effects, are widely used in medicine as anesthetics, rapid-acting antidepressants, and preclinical models of schizophrenia outcome measures. The results from this series of experiments provides some of the first evidence that AG and EGCG reduce the psychomotor stimulant and glutamate-releasing action of NMDA antagonists. Earlier studies with this behavioral model led to successful clinical trials of minocycline in the treatment of schizophrenia (Liu et al., 2014). Dissociative NMDA antagonists may also model dissociative, dysphoric, and anxiogenic symptoms of PTSD and delirium in medical patients. Our studies raise the possibility that other iNOS inhibitors such as AG and EGCG, which share with minocycline the ability to prevent microglial activation, may be beneficial in schizophrenia and delirium in medical patients. Nutraceuticals that counteract inflammatory nitric oxide release (green tea, carob powder and possibly black tea and chocolate) may also merit consideration as adjuncts to therapy.

## Author Contributions

Conceived and designed the experiments: SB and HB. Performed the experiments: HB and AD. Analyzed the data: SB and HB. Contributed reagents/materials/analysis tools: SB. Wrote the paper: SB, AD, and HB.

## Conflict of Interest Statement

The authors declare that the research was conducted in the absence of any commercial or financial relationships that could be construed as a potential conflict of interest.
