# Supplementary material for: Inducible Nitric Oxide Inhibitors Block NMDA Antagonist-Stimulated Motoric Behaviors and Medial Prefrontal Cortical Glutamate Efflux
Source: Front Pharmacol. 2015 Dec 15;6:292. doi: 10.3389/fphar.2015.00292 (PMC4678197; doi:10.3389/fphar.2015.00292)
Supplement: Supplementary file 1 [file Table_1.PDF]

Supplemental material

Rating scales for ataxia and stereotopy (adapted from Sturgeon *et al.*, 1979)

Rating Scale for Ataxia

0 – inactive or regular movement

1 – unusual, awkward, jerky movement, some loss of balance and rearing, 1 to 2 small falls

2 – moving and rearing, partially continuous falling or large falls

3 – continuous falling while moving, impaired antigravity reflexes

4 – movement contained to small area, supporting weight on haunches or abdomen, antigravity reflexes very impaired

5 – twitching or convulsive movements, rolling on side or raising head

Rating Scale for Stereotypy

0 – inactive or activity of a non-repetitive nature

1 – locomotor activity, sniffing or grooming

2 – weaving, non-directed movement, circling 1 or 2 times, more sniffing and rearing than in 1.

3 – turning, more circling, back-peddling, praying, more non-directed movement, sniffing and weaving

4 – rapid rate turning and continuous turning, back-peddling, praying, sniffing and weaving

5 – dyskinetic extension and flexion of limbs, head/neck and weaving
